# Inflammation in the Human Periodontium Induces Downregulation of the α_1_- and β_1_-Subunits of the sGC in Cementoclasts

**DOI:** 10.3390/ijms22020539

**Published:** 2021-01-07

**Authors:** Yüksel Korkmaz, Behrus Puladi, Kerstin Galler, Peer W. Kämmerer, Agnes Schröder, Lina Gölz, Tim Sparwasser, Wilhelm Bloch, Andreas Friebe, James Deschner

**Affiliations:** 1 Department of Periodontology and Operative Dentistry, University Medical Center of the Johannes Gutenberg University Mainz, 55131 Mainz, Germany; james.deschner@uni-mainz.de; 2 Department of Oral and Maxillofacial Surgery, University Hospital RWTH Aachen, RWTH Aachen University, 52074 Aachen, Germany; bpuladi@ukaachen.de; 3 Department of Conservative Dentistry and Periodontology, University Hospital Regensburg, 93042 Regensburg, Germany; kerstin.galler@ukr.de; 4 Department of Oral- and Maxillofacial and Plastic Surgery, University Medical Center Mainz, 55131 Mainz, Germany; peer.kaemmerer@unimedizin-mainz.de; 5 Department of Orthodontics, University Hospital Regensburg, 93053 Regensburg, Germany; agnes.schroeder@ukr.de; 6 Department of Orthodontics and Orofacial Orthopedics, University Hospital of Erlangen, Friedrich-Alexander University Erlangen-Nuernberg, 91054 Erlangen, Germany; lina.goelz@uk-erlangen.de; 7 Institute of Medical Microbiology and Hygiene, University Medical Center of the Johannes Gutenberg-University Mainz, 55131 Mainz, Germany; sparwasser.office@uni-mainz.de; 8 Department of Molecular and Cellular Sport Medicine, Institute of Cardiovascular Research and Sport Medicine, German Sport University Cologne, 50933 Cologne, Germany; w.bloch@dshs-koeln.de; 9 Institute of Physiology, University of Würzburg, 97070 Würzburg, Germany; andreas.friebe@uni-wuerzburg.de

**Keywords:** nitric oxide, soluble guanylyl cyclase, cGMP, cementoclasts, cementum, osteoclasts, alveolar bone, periodontitis

## Abstract

Nitric oxide (NO) binds to soluble guanylyl cyclase (sGC), activates it in a reduced oxidized heme iron state, and generates cyclic Guanosine Monophosphate (cGMP), which results in vasodilatation and inhibition of osteoclast activity. In inflammation, sGC is oxidized and becomes insensitive to NO. NO- and heme-independent activation of sGC requires protein expression of the α_1_- and β_1_-subunits. Inflammation of the periodontium induces the resorption of cementum by cementoclasts and the resorption of the alveolar bone by osteoclasts, which can lead to tooth loss. As the presence of sGC in cementoclasts is unknown, we investigated the α_1_- and β_1_-subunits of sGC in cementoclasts of healthy and inflamed human periodontium using double immunostaining for CD68 and cathepsin K and compared the findings with those of osteoclasts from the same sections. In comparison to cementoclasts in the healthy periodontium, cementoclasts under inflammatory conditions showed a decreased staining intensity for both α_1_- and β_1_-subunits of sGC, indicating reduced protein expression of these subunits. Therefore, pharmacological activation of sGC in inflamed periodontal tissues in an NO- and heme-independent manner could be considered as a new treatment strategy to inhibit cementum resorption.

## 1. Introduction

The intra- and intercellular signal molecule nitric oxide (NO) is synthesized by the activity of neuronal (n), endothelial (e), and inducible (i) isoforms of NO synthases (NOSs), which are active as homodimers [1,2]. NO-receptor enzyme soluble guanylyl cyclase (sGC) consists of two subunits (α and β) and has enzyme activity only in the heterodimeric α_1_β_1_- and α_2_β_1_-isoforms [3,4]. NO binds to the reduced iron (Fe^2+^) heme in the β_1_-subunit of sGC, resulting in activation of the heterodimeric α_1_β_1_- and α_2_β_1_-isoforms, which in turn leads to increased production of cyclic Guanosine Monophosphate (cGMP) from Guanosine-5′-Triphosphate (GTP) [3,4,5,6]. In the homodimer form, sGC has no enzyme activity [7,8]. The downstream effects of NO-cGMP signaling cascade are mediated through cGMP-dependent protein kinases (PKGs), cyclic nucleotide-gated channels (CNGs), and cGMP-regulated phosphodiesterases (PDEs) [4,9]. In addition to numerous functions such as smooth muscle relaxation, platelet aggregation inhibition, and neurotransmission [10], the NO-cGMP pathway also plays an important role in osteoblast-induced bone formation and osteoclast-dependent bone resorption [11,12].

Inflammation of the periodontium first occurs in the gingiva and can then gradually spread into the subgingival tissues, such as the periodontal ligament, cementum, and alveolar bone. If the periodontal inflammation persists or exaggerates, progressive bone destruction can follow as a result of increased osteoclastogenesis on the bone side of the periodontal ligament (PDL) space. Additionally, the inflammatory processes can promote cementoclastogenesis on the cementum side of the PDL [13,14,15,16]. The clast cells are multinucleated cells formed by the fusion of precursors of the monocyte lineage and are responsible for the resorption of bone, dentin, cementum, and mineralized cartilage [17,18]. The clast-induced resorption of hard tissues involves adhesion of the clast cells to the mineralized tissues, subsequent acidification of the area underneath the cells, and proteolytic degradation of the organic matrix by proteases [17,18,19,20,21,22]. 

NO induces differentiation of osteoclasts at lower concentrations and inhibits differentiation of osteoclasts at higher concentrations [23,24]. The treatment of mature osteoclasts with NO donors inhibits resorptive osteoclastic activity [25,26]. NO-independent sGC activators (e.g., YC-1) inhibit osteoclast differentiation, indicating that sGC may have an inhibitory effect on osteoclast differentiation and bone resorption via cGMP and PKG [11,12,26,27]. However, under inflammatory conditions, sGC is oxidized (Fe^3+^) due to the increased presence of reactive oxygen species (ROS) and reactive nitrogen species (RNS) and, therefore, exists in an NO-insensitive state [6]. Activators, such as the NO- and partially heme-independent sGC activator YC-1 [28] and the NO- and heme-independent activator of sGC cinaciguat (BAY 58-2667) [5] were developed to increase the activity of sGC in diseased tissues. 

In periodontitis, sustained activation of iNOS produces higher amounts of NO [29,30,31,32], which in turn promotes alveolar bone resorption [33,34]. ROS have also been reported to cause alveolar bone loss [35,36]. It is expected that sGC is oxidized by RNS and ROS in the inflamed periodontium and therefore may be present in a NO-insensitive state in cementoclasts and osteoclasts. The sGC activation by YC-1 and BAY 58-2667 in osteoclasts and cementoclasts under inflammatory conditions requires protein expression of the α_1_- and β_1_-subunits in these cells. Therefore, clarifying the presence of heterodimeric α_1_β_1_- and α_2_β_1_-isoforms of sGC in cementoclasts is important for possible pharmacological treatment of the inflamed periodontium. In a previous study, we have shown nNOS, eNOS, and its phosphorylated form at Ser1177, the α_2_- and β_1_-subunits of sGC, and cGMP in osteoclasts of rat alveolar bone [37]. However, whether the α_1_- and β_1_-subunits of sGC are present in cementoclasts of the human periodontium under healthy and inflamed conditions is not known and deserves further clarification. Therefore, the objective of the study was to examine the α1- and β1-subunits of sGC in cementoclasts of healthy and inflamed human periodontium by quantitative and double immunohistochemical methods.

## 2. Results

### 2.1. Characterization of the Healthy Periodontium and Presence of the α_1_- and β_1_-Subunits of sGC in Cementoclasts

**Figure 1:**
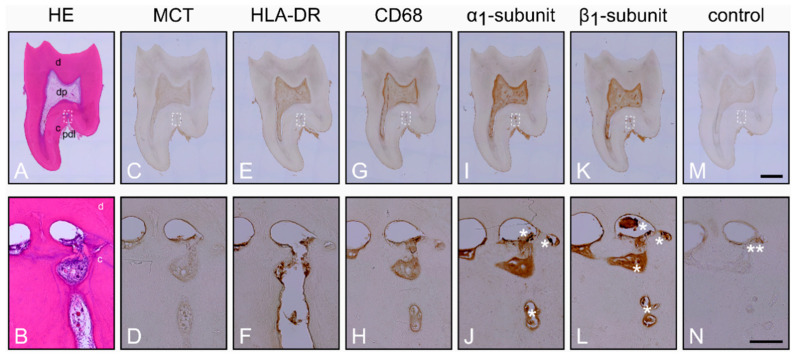
Histological characterization of the healthy periodontium and localization of the α_1_- and β_1_-subunits of guanylyl cyclase (sGC) in cementoclasts: consecutive sections of a representative molar with adherent clinically healthy periodontium were stained with Hematoxylin and Eosin (HE) (**A**,**B**) and incubated with antibodies for Mast Cell Tryptase (MCT) (**C**,**D**), Human Leukocyte Antigen-DR isotype (HLA-DR) (**E**,**F**), CD68 (**G**,**H**), α_1_-subunit (**I**,**J**), and β_1_-subunit (**K**,**L**), respectively. Incubation of sections without primary antibodies served as control (**M**,**N**). The images of the upper row show an overview of the consecutive sections, while the detail images of the lower row show the regions represented by the rectangle in the overview images of the upper row. d = dentin, dp = dental pulp, c = cementum, pdl = periodontal ligament. Asterisks in (**J**) and (**L**) shown cementoclasts in resorption lacunae, while asterisks in (**N**) only show an artifact. Scale bar = 2 mm (**M**) and = 200 µm (**N**).

First, we sought to examine whether the α_1_- and β_1_-subunits of sGC are expressed in human cementoclasts. Therefore, caries-free molars with clinically healthy periodontium and carious molars with clinically inflamed periodontal tissues were collected from patients. As assessed with Hematoxylin and Eosin (HE), healthy periodontium of the caries-free molars was characterized by a regular form and structure (Figure 1A). In PDL, the blood vessels were intact and numerous structural cells with normal appearance and regular distribution were detectable. In some healthy molars with periodontium, resorption lacunae of varying size and number were observed in the transition region from PDL to cementum, in the cementum, and in the transition region from the cementum to dentin (Figure 1A,B; Figure S1 in Supplementary Materials).

Further characterization was performed for mast cells (MCT), monocytes, dendritic cells, lymphocytes (HLA-DR) and macrophages (CD68) by using avidin-biotin peroxidase complex. While mast cell tryptase (Figure 1C,D) was not detectable in the healthy periodontium, HLA-DR (Figure 1E,F) was expressed. Interestingly, the absence of mast cell tryptase (Figure 1C,D) and the presence of HLA-DR (Figure 1E,F) was also observed in dental pulp tissue. Moreover, CD68 was also found in cells of the healthy periodontium and dental pulp (Figure 1G,H). In the following consecutive sections, α_1_- (Figure 1I,J) and β_1_-subunits (Figure 1K,L) of sGC were detected with strong staining intensities in cementoclasts located in resorption lacunae of the cementum. The expression of the α_1_- (Figure 1I,J) and β_1_-subunits (Figure 1K,L) in pulpal blood vessels and odontoblasts served as a positive control for the antibodies used [38,39]. Incubation of the consecutive section without primary antibodies did not result in immunohistochemical staining (Figure 1M,N).

### 2.2. Characterization of the Inflamed Periodontium and Presence of the α_1_- and β_1_-Subunits of sGC in Cementoclasts

**Figure 2:**
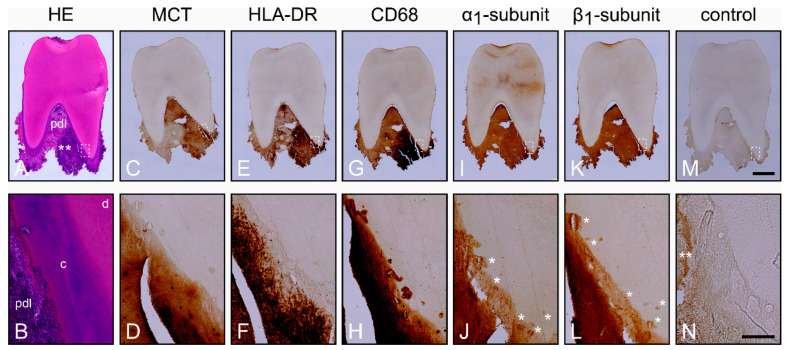
Histological characterization of the inflamed periodontium and localization of the α_1_- and β_1_-subunits of sGC in cementoclasts. Consecutive sections of a representative molar with adherent clinically inflamed periodontium were stained with Hematoxylin and Eosin (HE) (**A**,**B**) and incubated with antibodies for MCT (**C**,**D**), HLA-DR (**E**,**F**), Cluster of Differentiation 68 (CD68) (**G**,**H**), α_1_-subunit (**I**,**J**), and β_1_-subunit (**K**,**L**). Incubation of the sections without primary antibodies served as a control (**M**,**N**). The images in the upper row show an overview of the consecutive sections, while the detailed images in the lower row show the regions represented by the rectangle in the overview images of the upper row. d = dentin, c = cementum, and pdl = periodontal ligament. Asterisks in (**J**) and (**L**) shown cementoclasts in resorption lacunae, while asterisks in (**N**) only show an artifact. Scale bar = 2 mm (**M**) and = 200 µm (**N**).

In contrast to the healthy periodontium, numerous inflammatory cells were detected in the PDL of inflamed periodontium, as assessed with HE (Figure 2A,B; Figure S1). In some regions, inflammatory cells were seen in the PDL and in the transition region from PDL to cementum (Figure 2A,B). The blood vessels were destructured due to the severe inflammation, and the PDL contained a large number of acute and chronic inflammatory cells (Figure 2A). As compared to healthy periodontium, resorption lacunae at higher number and sometimes also at greater size were mainly found in the transition region from PDL to cementum. Further immunohistochemical analyses revealed a remarkable staining for MCT and therefore numerous mast cells in the inflamed PDL (Figure 2C,D), which was in strong contrast to the healthy periodontium. 

HLA-DR was highly expressed in immune cells of the inflamed PDL (Figure 2E,F). The immunostaining for Cluster of Differentiation 68 (CD68) was more pronounced in the inflamed PDL, indicating increased numbers of macrophages and cementoclasts (Figure 2G,H). Although, in the following consecutive sections, α_1_- (Figure 2I,J) and β_1_-subunits (Figure 2K,L) of sGC were detected in cementoclasts of the resorption lacunae of the cementum, the staining intensities were lower than in the healthy periodontium. Incubation of the consecutive section without primary antibodies did not lead to immunohistochemical staining (Figure 2M,N).

### 2.3. The Colocalization of α_1_- and β_1_-Subunits of sGC with CD68 in Cementoclasts of Inflamed Human Periodontium

**Figure 3:**
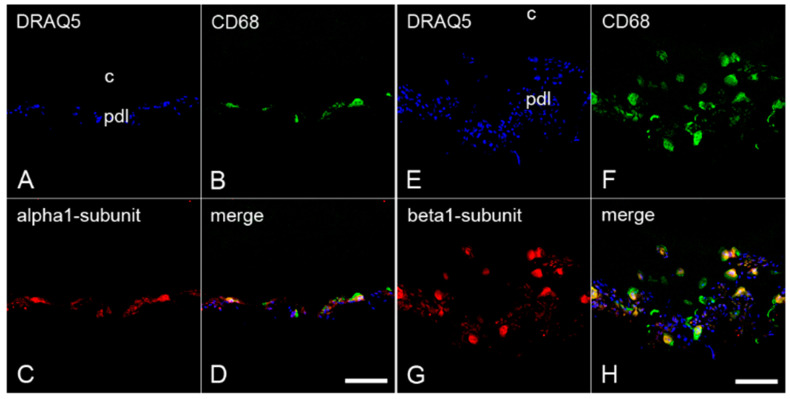
Colocalization of the α_1_- and β_1_-subunits of sGC with CD68 in cementoclasts of inflamed human periodontium: sections of a representative molar with adherent clinically inflamed periodontium were stained with Deep Red Anthraquinone 5 (DRAQ5) (**A**,**E**) and incubated with antibodies for CD68 (**B**,**F**), α_1_-subunit (**C**), and β_1_-subunit (**G**) of sGC. Colocalization of CD68 with the α_1_-subunit (**D**) and β_1_-subunit (**H**) in cementoclasts is depicted in the merged images. c = cementum, and pdl = periodontal ligament. Scale bar = 100 µm (**D**,**H**).

**Figure 4:**
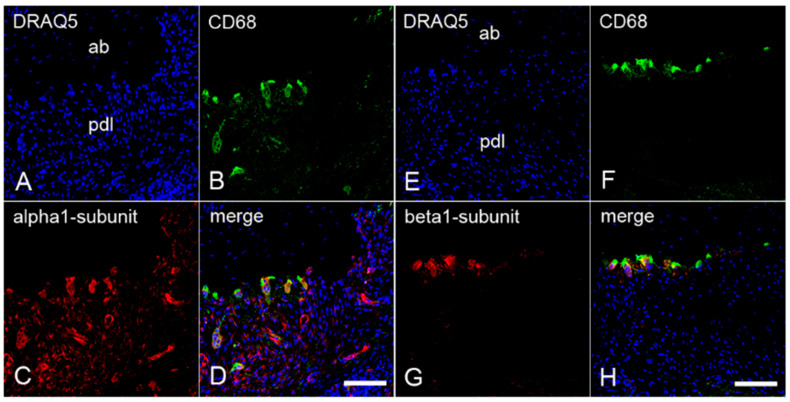
Colocalization of the α_1_- and β_1_-subunits of sGC with CD68 in osteoclasts of inflamed human periodontium: sections of a representative molar with adherent clinically inflamed periodontium were stained with DRAQ5 (**A**,**E**) and incubated with antibodies for CD68 (**B**,**F**), α_1_-subunit (**C**), and β_1_-subunit (**G**) of sGC. Colocalization of CD68 with the α_1_-subunit (**D**) and β_1_-subunit (**H**) in osteoclasts is depicted in the merged images. ab = alveolar bone, and pdl = periodontal ligament. Scale bar = 100 µm (**D**,**H**).

To demonstrate the possible presence of α_1_- and β_1_-subunits of sGC in cementoclasts and osteoclasts, colocalization of these subunits with CD68 was investigated by immunofluorescence double staining. To test the colocalization, sections of the inflamed periodontium containing both cementoclasts and osteoclasts were incubated with an antibody against CD68 and an antibody against α_1_-subunit or β_1_-subunit, respectively. On the cementum side of the inflamed human periodontium, CD68 was colocalized with the α_1_-subunit (Figure 3A–D) and the β_1_-subunit (Figure 3E–H) of sGC in cementoclasts. Similarly, on the alveolar bone side of the inflamed periodontium, colocalization of CD68 with the α_1_-subunit (Figure 4A–D) and the β_1_-subunit (Figure 4E–H) of sGC was found in osteoclasts.

### 2.4. The Colocalization of α_1_- and β_1_-Subunits of sGC with Cathepsin K in Cementoclasts of Inflamed Human Periodontium

**Figure 5:**
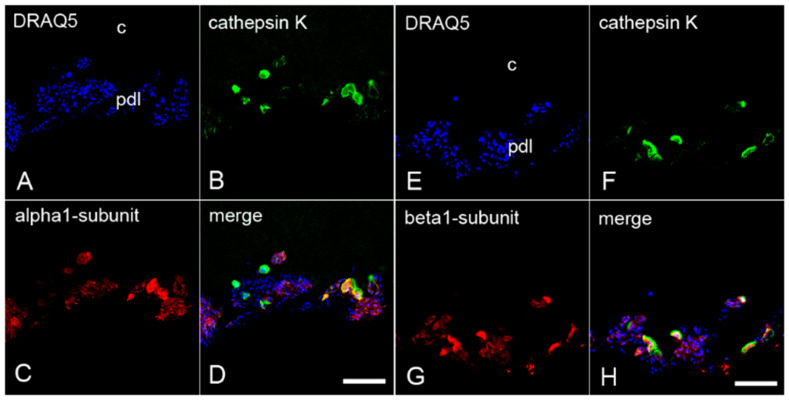
Colocalization of the α_1_- and β_1_-subunits of sGC with cathepsin K in cementoclasts of inflamed human periodontium: sections of a representative molar with adherent clinically inflamed periodontium were stained with DRAQ5 (**A**,**E**) and incubated with antibodies for cathepsin K (**B**,**F**), α_1_-subunit (**C**), and β_1_-subunit (**G**) of sGC. Colocalization of cathepsin K with the α_1_-subunit (**D**) and β_1_-subunit (**H**) in cementoclasts is depicted in the merged images. c = cementum, and pdl = periodontal ligament. Scale bar = 100 µm (**D**,**H**).

**Figure 6:**
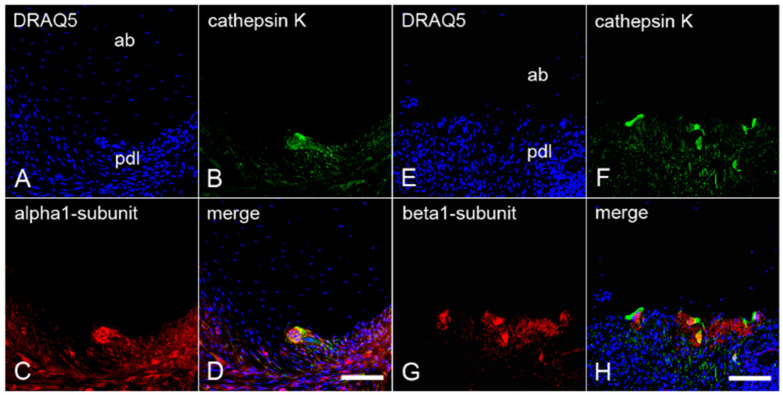
Colocalization of the α_1_- and β_1_-subunits of sGC with cathepsin K in osteoclasts of inflamed human periodontium: sections of a representative molar with adherent clinically inflamed periodontium were stained with DRAQ5 (**A**,**E**) and incubated with antibodies for cathepsin K (**B**,**F**), α_1_-subunit (**C**), and β_1_-subunit (**G**) of sGC. Colocalization of cathepsin K with the α_1_-subunit (**D**) and β_1_-subunit (**H**) in osteoclasts is depicted in the merged images. ab = alveolar bone, and pdl = periodontal ligament. Scale bar = 100 µm (**D**,**H**).

To confirm the presence of both sGC subunits in cementoclasts and osteoclasts of human inflamed periodontium, sections containing both cells types were incubated with an antibody against α_1_-subunit or β_1_-subunit, and cathepsin K. Afterwards, immunofluorescence double staining was performed again. Cathepsin K was colocalized with the α_1_- subunit (Figure 5A–D) and the β_1_-subunit (Figure 5E–H) of sGC in cementoclasts on the cementum side. In addition, cathepsin K was colocalized with the α_1_-subunit (Figure 6A–D) and the β_1_-subunit (Figure 6E–H) in osteoclasts on the alveolar bone side of the inflamed periodontium.

### 2.5. The Staining Intensities of α_1_- and β_1_-Subunits of sGC in Cementoclasts of the Healthy and Inflamed Human Periodontium

**Figure 7:**
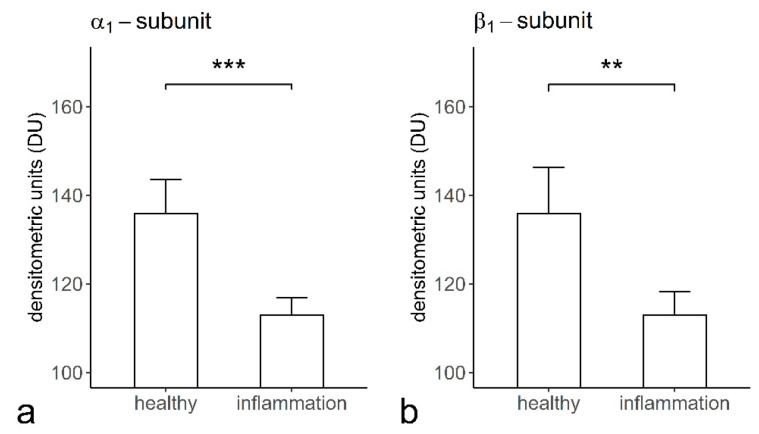
The staining intensities of the α_1_- and β_1_-subunits of sGC in cementoclasts of the healthy and inflamed human periodontium: staining intensity of the α_1_-subunit in cementoclasts, as assessed in sections of healthy (*n* = 6) and inflamed (*n* = 6) periodontium by densitometric analysis (**a**), and staining intensity of the β_1_-subunit in cementoclasts, as analyzed in sections of healthy (*n* = 6) and inflamed (*n* = 6) periodontium by densitometric analysis (**b**). Data are presented as mean ± standard deviation (SD). The significant differences between two groups were presented with an asterisk (**a**, *** *p* < 0.001; **b**, ** *p* < 0.01).

The staining intensity of the α_1_- (135.79 ± 07.81 densitometrical unit (DU)) (Figure 7A) and β_1_- (136.03 ± 10.39 DU) (Figure 7B) subunits of sGC in cementoclasts of the healthy periodontium was significantly higher than that of the α_1_- (112.87 ± 03.94 DU) (Figure 7A) and β_1_- (113.17 ± 05.46 DU) (Figure 7B) subunits of sGC in cementoclasts of the inflamed periodontium. 

## 3. Discussion

**Figure 8:**
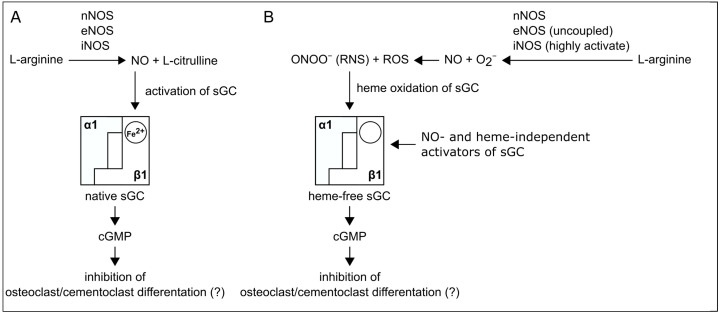
Schematic illustration of the possible role of sGC in the NO-cGMP signaling cascade in osteoclasts and cementoclasts of the human periodontium (modified from [44]): (**A**) In osteoclasts and cementoclasts of healthy periodontium, nitric oxide (NO) is synthesized from the amino acid L-arginine by activated neuronal (n), endothelial (e), and/or inducible (i) NO synthase (NOS). At physiological concentrations, NO binds to the heme of the β1-subunit of soluble guanylyl cyclase (sGC), thereby activating the heterodimeric α1β1-isoform, which leads to increased production of cGMP from GTP. This could result in inhibition of osteoclast and cementoclast differentiation. (**B**) In osteoclasts and cementoclasts of the inflamed periodontium, the β1-subunit of sGC is oxidized (Fe^3+^) by the formation of reactive oxygen species (ROS) and reactive nitrogen species (RNS) and therefore exists in a NO-insensitive state. Nevertheless, as in healthy periodontium, sGC is also present in osteoclasts and cementoclasts in inflamed periodontium at the protein level. Inactivation of sGC may lead to increased osteoclast and cementoclast differentiation in periodontitis. Pharmacological activation of the α1β1-isoform of sGC in cementoclasts and osteoclasts by NO- and heme-independent activators of sGC could therefore be considered a novel treatment strategy to inhibit both cementum and alveolar bone resorption [12,45].

Inflammation of the periodontium induces the resorption of cementum by cementoclasts and the resorption of the alveolar bone by osteoclasts. Previously, it has been reported that sGC has an inhibitory effect on osteoclast differentiation and bone resorption [11,12,26,27]. Furthermore, it has been demonstrated that sGC is also expressed in osteoclasts [37]. However, whether sGC is also produced by cementoclasts of healthy and inflamed periodontium was unknown and therefore investigated in the present study. In comparison to cementoclasts in the healthy periodontium, cementoclasts under inflammatory conditions showed a decreased staining intensity for both α_1_- and β_1_-subunits of sGC, indicating reduced protein expression of these subunits. Therefore, pharmacological activation of sGC in inflamed periodontal tissues in an NO- and heme-independent manner could be considered a new treatment strategy to inhibit cementum resorption (Figure 8) [12,27].

For development of new strategies for the treatment of periodontal inflammation and therefore destruction, a fundamental understanding of the functional characteristics of cementoclasts and osteoclasts under normal and inflammatory conditions is critical. In this context, clarification of the existence and role of sGC in cementoclasts and osteoclasts under the aforementioned conditions could provide new insights into the regulatory mechanisms of alveolar bone and cementum resorption. Whether sGC is produced identically or differently in different types of clast cells, e.g., cementoclasts and osteoclasts, is also of importance. Our results show that the α_1_- and β_1_-subunits of sGC were similarly expressed in both cementoclasts and osteoclasts. In addition, their expressions in these cells were similarly regulated by inflammation, i.e., the periodontal inflammatory processes led to a decrease in the protein expression of both subunits. Since the α_1_- and β_1_-subunits of sGC were detectable in the cementoclasts, our results indicate that sGC is present in the active α_1_β_1_-isoform. However, additional in vitro and in vivo experiments are required to clarify which functions the α_1_β_1_-isoform of sGC can fulfill in cementoclasts.

The resorption of cementum is characterized with the formation of resorption lacunae by activated cementoclasts. This process leads in turn to the activation of cementoblasts, which then fill the resorption lacunae by depositing cementum. It is known that forces generated by occlusal trauma or orthodontic treatment trigger a sterile inflammatory process that can activate cementoclasts to resorb cementum [40,41]. Since the molars of the healthy periodontium were not subjected to orthodontic forces, the occurrence of cementoclasts located in resorption lacunae, as observed in our study, could have been the result of physiological and/or traumatic occlusal forces.

Under physiological conditions, we have shown the expression of α_1_- and β_1_-subunits of sGC in cementoclasts. In a healthy state, the β_1_-subunit of sGC may contain reduced iron (Fe^2+^) heme and, therefore, it may be activated by physiological concentrations of endogenous NO. It has been reported that sGC exerts inhibitory effects on osteoclast activity [11,12,26,27]. In mature osteoclasts, NO causes activation of sGC and therefore formation of cGMP, which leads to inhibition of osteoclast adhesion and acid secretion [42,43]. Therefore, NO-induced activation of sGC under physiological conditions ensures a balance between cementum resorption and formation. 

However, inflammation of the periodontium can disturb the balance between cementoblasts and cementoclasts [13,16]. Since periodontal inflammation leads to an increased activity of cementoclasts on the root side and osteoclasts on the alveolar bone side while at the same time the activity of cementoblasts and osteoblasts is inhibited, the resorption lacunae of the cementum and alveolar bone cannot be filled up. As a result, there is loss of mineralized and soft tissues, which can ultimately lead to increased tooth mobility and loss. Under inflammatory conditions, we detected the expression of the α_1_β_1_-isoform of sGC in both cementoclasts and osteoclasts at the protein level. In inflammation, sGC is oxidized (Fe^3+^) and subsequently heme-free [6,46]. Heme oxidation of the β_1_-subunit of sGC triggers ubiquitination and subsequent proteolytic degradation of both α_1_- and β_1_-subunits of sGC [47]. Ubiquitination of sGC may be prevented by the heme-binding site ligand BAY 58-2667, an sGC activator, that stabilizes the α_1_- and β_1_-subunits of sGC [47]. Under inflammatory conditions, the β_1_-subunit of sGC is oxidized (Fe^3+^) and sGC is therefore present in an NO-insensitive state [6]. In osteoporosis, sGC is also insensitive to NO due to the excessive formation of ROS and RNS [45,48]. In patients with osteoporosis, treatment strategies have been developed to inhibit excessive bone resorption and to increase bone formation [12,49]. To inhibit excessive bone resorption and to enhance bone formation via NO-cGMP signaling, NO- and heme-independent sGC activators can be used in the treatment of osteoporosis [12,45]. Thus, NO- and heme-independent activators of sGC may have great potential for the treatment of an inflamed periodontium. However, whether BAY 58-2667 is able to stabilize the α_1_- and β_1_-subunits of sGC in cementoclasts and osteoclasts of the inflamed human periodontium has to be unraveled in future in vitro and clinical studies. Therefore, future in vitro studies may help to unravel the underlying regulatory mechanisms.

In summary, we provide original evidence that both α_1_- and β_1_-subunits of sGC are produced in cementoclasts of the human periodontium. Furthermore, we confirm the presence of both sGC subunits in osteoclasts. Moreover, we show for the first time that the expression of the α_1_- and β_1_-subunits in cementoclasts and osteoclasts is reduced in the inflamed human periodontium. Since sGC has been shown to have an inhibitory effect on osteoclasts, pharmacological activation of sGC in inflamed periodontal tissues could be considered a new treatment strategy that involves inhibition of cementum resorption (Figure 8).

## 4. Materials and Methods

### 4.1. Tissue Sample Collection

Caries-free molars with healthy periodontal tissues (*n* = 25) and carious molars with inflamed periodontium (*n* = 13) were extracted for orthodontic reasons. Immediately after extraction, the molars were immersion-fixed in a fixative containing 4% paraformaldehyde and 0.2% picric acid in 0.1 M phosphate-buffered saline (PBS), pH 7.4, for 24 h and demineralized in 4 M formic acid for 21 days. The molars with periodontal tissues were cryoprotected with 30% sucrose solution in 0.1 M PBS, pH 7.4, for 48 h; frozen-embedded; stored at −82 °C; and frozen-sectioned on a cryostat at 30 µm. 

### 4.2. Histological Evaluation of the Inflammatory State

Whereas the molars of the healthy group were unrestored and clinically asymptomatic and had no pain on percussion, molars of the inflamed group showed spontaneous or percussion pain, clinical symptoms, and/or radiographic periodontal alteration. The sections of molars with adherent periodontium were stained with Hematoxylin and Eosin (HE). In a subgroup of teeth, additional immunohistochemical staining for mast cell tryptase (MCT), HLA-DR (monocytes, dendritic cells, and activated T and B cells marker), and CD68 (macrophage marker) was performed to characterize the leukocyte types in the healthy and inflamed periodontium. 

### 4.3. The Specificity of Antibodies against Human α_1_- and β_1_-Subunits of sGC

To detect the α_1_-subunit of sGC in cementoclasts and osteoclasts, we developed a specific polyclonal rabbit antibody against the human α_1_-subunit of sGC (EP101278: ID0490; Eurogentec, Seraing, Belgium) that was purified by peptide affinity chromatography and characterized by ELISA, immunohistochemistry, and immunoblotting [38]. The specificity of the polyclonal rabbit antihuman β_1_-subunit of sGC antibody was also previously developed by our group and tested by immunoblotting using lung protein extracts of the sGCβ_1_^+/+^ and sGCβ_1_^−/−^ mice as described previously [39]. 

### 4.4. Immunohistochemical Avidin-Biotin-Peroxidase Complex Method

The free-floating sections in 12-well plates were first treated with 0.3% H_2_O_2_ in 0.05 M Tris-buffered saline (TBS) for 20 min. The sections were incubated with 0.25% Triton X-100 in 0.6 M TBS, pH 7.6, for 30 min. The nonspecific immunoglobulin binding sites were blocked by incubation of the sections with a blocking solution containing 5% normal goat serum (Vector, Burlingame, CA, USA) and 2% bovine serum albumin (Sigma-Aldrich, Taufkirchen, Germany). The consecutive sections were incubated with mouse antihuman cathepsin K (Santa Cruz Biotechnology, Santa Cruz, CA, USA), mouse antihuman MCT (Santa Cruz Technology, Santa Cruz, CA, USA), mouse antihuman CD68 (eBioscience, San Diego, CA, USA), mouse antihuman HL-DR (eBioscience, San Diego, CA, USA), and polyclonal rabbit antihuman α_1_- (1:1000) [38] and β_1_-subunits (1:1000) [39] antibodies at 4 °C. The sections were incubated with biotinylated goat anti-mouse or goat anti-rabbit IgG (1:500) (Vector) for 1 h, respectively, and subsequently with avidin-biotin peroxidase complex (1:100) (Vector) for 1 h. The immunohistochemical reaction was developed in all sections with 0.05% 3,3’-diaminobenzidine tetrahydrochloride (Sigma-Aldrich, Taufkirchen, Germany) in 0.05 M Tris-HCl buffer, pH 7.6, containing 0.01% H_2_O_2_ and 0.01% nickel sulfate for 15 min [38,50].

### 4.5. Immunofluorescence Double Staining Method

The free-floating sections in 12-well plates were incubated with 5% normal goat serum to block the nonspecific immunoglobulin binding sites of the secondary antibodies. The sections were incubated first with antibodies against mouse antihuman CD68 (eBioscience) and mouse antihuman cathepsin K (Santa Cruz Biotechnology, Santa Cruz, CA, USA) at 4 °C. Then, the sections were incubated with DyLight^TM^ 488-conjugated goat anti-mouse IgG (Thermo Fischer Scientific, Waltham, MA, USA) for 1 h. The sections were then incubated with rabbit polyclonal antihuman α_1_-subunit (1:1000) [38] and β_1_-subunit (1:1000) [39] antibodies overnight at 4 °C in the dark. Thereafter, the sections were incubated for 1 h with the DyLight^TM^ 550-conjugated goat anti-rabbit IgG (Thermo Fischer Scientific). To show the cell nuclei, sections were stained with Hoechst 33342 and DRAQ5 (Cell Signaling Technology, Frankfurt am Main, Germany) for immunofluorescence microscopy and LSM confocal microscopy, respectively, for 15 min. The sections were coverslipped with Aqua Poly/Mount (Polysciences Inc., Warrington, PA, USA) and analyzed with an LSM510 confocal microscope (Carl Zeiss, Jena, Germany) [50].

### 4.6. Controls

Since we had previously detected α_2_- and β_1_-subunits of sGC in osteoclasts of alveolar bone [37], sections containing both cementum and alveolar bone which served as a positive control for sGC were selected. To control immunohistochemical reagents (secondary antibodies and avidin-biotin peroxidase complex), sections were also incubated in the absence of the primary antibodies. 

### 4.7. Quantification of Staining Intensities of α_1_- and β_1_-Subunits and Statistical Analysis

The staining intensities were measured in section-free regions (background grey value) and in cementoclasts (cementoclast grey value) stained for the α_1_- and β_1_-subunits in consecutive sections. Immunostaining intensity for healthy (*n* = 6) and inflamed (*n* = 6) human periodontia from 6 individuals per group was calculated as the background grey value minus the mean of the selected cementoclast grey value [38,50]. 

Statistical analysis was performed using IBM SPSS Statistics version 27.0 (SPSS, Inc., Chicago, IL, USA). Statistical significances of the differences were determined by using the unpaired Student *t* test. A *p* value < 0.01 was considered statistically significant. The ggplot2 package was applied for data visualization.

## 5. Conclusions

The α_1_- and β_1_-subunits of sGC were expressed at the protein level in cementoclasts of healthy and inflamed human periodontium. In periodontal inflammation, increased oxidative and nitrogen stress may induce desensitization of the β1-subunit of sGC in cementoclasts and osteoclasts. Therefore, pharmacological activation of the α_1_β_1_-isoform of sGC in cementoclasts and osteoclasts by sGC activators could be considered a new treatment strategy to inhibit both cementum and alveolar bone resorption. 

## Data Availability

Data sharing is not applicable to this article.

## Supplementary Materials

The following are available online at https://www.mdpi.com/1422-0067/22/2/539/s1.

## Abbreviations

HE	Hematoxylin and Eosin
NO	Nitric Oxide
NOS	Nitric Oxide Synthase
sGC	soluble Guanylyl Cyclase
cGMP	cyclic Guanosine Monophosphate
GTP	Guanosine-5′-Triphosphate
PKG	protein kinase G
CNG	cyclic nucleotide-gated channel
PDE	phosphodiesterase
PDL	periodontal ligament
MCT	Mast Cell Tryptase
HLA-DR	Human Leukocyte Antigen-DR isotype
CD68	Cluster of Differentiation 68
DRAQ5	Deep Red Anthraquinone 5
ROS	Reactive Oxygen Species
RNS	Reactive Nitrogen Species
