# Supplementary material for: Inflammation in the Human Periodontium Induces Downregulation of the α1- and β1-Subunits of the sGC in Cementoclasts
Source: Int J Mol Sci. 2021 Jan 7;22(2):539. doi: 10.3390/ijms22020539 (PMC7827426; doi:10.3390/ijms22020539)
Supplement: Supplementary file 1 [file ijms-22-00539-s001.pdf]

Supplementary Materials:

Yüksel Korkmaz[1,*], Behrus Puladi[2], Kerstin Galler[3], Peer W. Kämmerer[4], Agnes Schröder[5], Lina Gölz[6], Tim Sparwasser[7], Wilhelm Bloch[8], Andreas Friebe[9], James Deschner[1]

[1] Department of Periodontology and Operative Dentistry, University Medical Center of the Johannes Gutenberg University Mainz, Augustusplatz 2, 55131 Mainz, Germany; yueksel.korkmaz@unimedizin-mainz.de; james.deschner@uni-mainz.de

[2] Department of Oral and Maxillofacial Surgery, University Hospital RWTH, RWTH Aachen University, Aachen, Germany; bpuladi@ukaachen.de

[3] Department of Conservative Dentistry and Periodontology, University Hospital Regensburg, Regensburg, Germany; kerstin.galler@ukr.de

[4] Department of Oral- and Maxillofacial and Plastic Surgery, University Medical Center Mainz, Augustusplatz 2, 55131 Mainz, Germany; peer.kaemmerer@unimedizin-mainz.de

[5] Department of Orthodontics, University Hospital Regensburg, Franz Josef Strauss Allee 11, 93053 Regensburg, Germany; agnes.schroeder@ukr.de

[6] Department of Orthodontics and Orofacial Orthopedics, University Hospital of Erlangen, Friedrich-Alexander University Erlangen-Nuernberg, Glueckstr. 11, 91054 Erlangen, Germany; lina.goelz@uk-erlangen.de

[7] Institute of Medical Microbiology and Hygiene, University Medical Center of the Johannes Gutenberg-University Mainz, Mainz, Germany; sparwasser.office@uni-mainz.de

[8] Institute of Cardiovascular Research and Sport Medicine, Department of Molecular and Cellular Sport Medicine, German Sport University Cologne, Am Sportpark Muengersdorf 6, Cologne 50933, Germany; w.bloch@dshs-koeln.de

[9] Institute of Physiology, University of Würzburg, Röntgenring 9, D-97070, Würzburg, Germany; andreas.friebe@uni-wuerzburg.de

* Correspondence: yueksel.korkmaz@unimedizin-mainz.de; Tel.: 0049 6131 17-7247

Figure S1

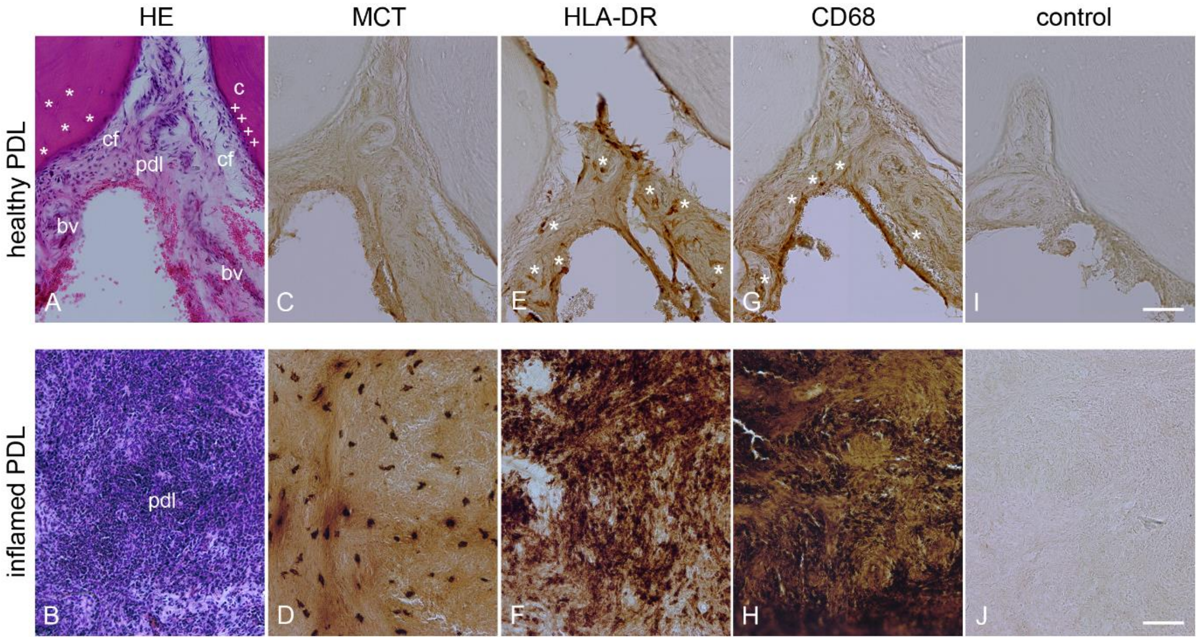

Figure S1. Histologic characterization of healthy and inflamed human periodontium by HE staining and immunohistochemical analysis for MCT, HLA-DR, and CD68 in consecutive sections. In the HE-stained healthy PDL, numerous cells with blue-stained nuclei were identified (A). In the healthy PDL, collagen fibers (cf) and blood vessels (bv) with numerous erythrocytes were clearly visible (A). The cementum contained cementocytes (asterisks), whereas cementoblasts (plus characters) were found in the transition area from the PDL to the cementum (A). In the HE-stained, severely inflamed PDL, structural ordering of the tissue was not evident (B). Dense lymphocytic infiltrates were observed in various regions of the inflamed PDL (B). The blood vessels were severely degraded (B). In the consecutive section from the healthy PDL, no MCT could be identified (C), whereas numerous MCT-positive cells were detected in the chronically inflamed PDL (D). In the following section from the healthy PDL, HLA-DR was found in some cells (asterisks; E), whereas stronger staining for HLA-DR was detected in numerous cells from the inflamed PDL (F). Similarly, CD68 was found in some cells of healthy PDL (asterisks; G). A strong increase in staining intensity for CD68 was detected in the inflamed PDL (H). No staining was found in consecutive sections of the healthy (I) and inflamed PDL (J) in which the primary antibodies were omitted as controls. Scale bar= 100 µm.
